# Role of Cue Training, Context, and Stimulus Intensity on Fear Generalization in Humans

**DOI:** 10.3390/bs13060479

**Published:** 2023-06-07

**Authors:** Yu Gao, Shaochen Zhao, Zifan Yang, Haote Fu, Keying Luo, Wei Chen, Min Fan, Yidan Song, Xifu Zheng

**Affiliations:** 1 Key Laboratory of Brain, Cognition and Education Sciences, Ministry of Education, South China Normal University, Guangzhou 510631, China; 2 School of Psychology, Center for Studies of Psychological Application and Guangdong Key Laboratory of Mental Health and Cognitive Science, South China Normal University, Guangzhou 510631, China; 3 Research Center for Guangdong-HongKong-Marcao Policing Model Innovation, China People’s Police University, Guangzhou 510663, China

**Keywords:** discriminative learning, cognitive rules, contextual generalization, stimulus intensity, generalization gradient

## Abstract

Fear generalization is a crucial mechanism underlying maladaptive behavior, but factors influencing this process are not fully understood. We investigated the effects of cue training and context on fear generalization and how cognitive rules influence responses to different conditions. We also examined the role of stimulus intensity in fear generalization to provide insight into fear generalization mechanisms. Participants (*n* = 104) completed a fear emotion task with two stages: acquisition and generalization testing. Subjective fear expectancy ratings were used as outcome measures. Participants who received single threat cue training exhibited stronger fear generalization responses than those who received discrimination training with threat and safe cues. Participants who received discrimination training and used linear rules had the strongest fear response to the largest stimulus. Therefore, a safe cue may mitigate fear generalization but could increase fear responses to more intense stimuli. Altering context did not change the fear generalization response because fear generalization is mainly governed by the association between the conditioned stimulus and the unconditioned fear stimulus. The present study emphasizes the multifaceted nature of fear generalization and the importance of examining multiple factors to understand this phenomenon. These findings elucidate fear learning and provide insights needed for effective interventions for maladaptive behavior.

## 1. Introduction

Fear is an emotional experience that usually arises from the recognition of the existence of danger or threat. As a natural survival response, fear helps individuals cope with potential dangers [1]. Fear generalization is the development of similar fear responses in individuals facing situations that are similar but not identical to previous threats or fearful situations because of the influence of prior experiences [2,3]. Reducing the threshold for reacting to potential threats is appropriate for adaptation and survival, but excessive reduction may impair adaptive behavior and result in high-cost avoidance behaviors [4,5]. Therefore, achieving a balance between safety and adaptive needs of fear generalization is critical. Understanding the perception and cognitive processing of stimuli and contexts may advance the understanding of fear generalization, which is needed to promote adaptive emotional responses.

### 1.1. Effects of Cue Training on Fear Generalization

Classical conditioning was an early method used to study fear generalization. By pairing a neutral conditioned stimulus (CS) with an unconditioned stimulus (US), individuals acquire a conditioned fear response to the original neutral stimulus. The development of fear expectations in response to the presence of a similar but innocuous new stimulus indicates the result of fear generalization. These new stimuli are referred to as generalization stimuli (GS) [2].

In the single cue fear conditioning paradigm, only one reinforced stimulus was presented along with the US as the CS+, resulting in the acquisition of an association between the single stimulus and the US. At this point, individuals have a stronger fear response to the CS+ [6]. As the similarity between the GS and the CS+ decreases, the fear response to the GS weakens [3,7]. In the discriminative fear conditioning paradigm, individuals were required to learn different conditional stimuli: one was associated with the US as the threat cue (CS+), and the other was not associated with the US as the safety cue (CS−). Individuals need to learn the difference in the CS-US association between different stimuli, resulting in a stronger fear response to the CS+ and a lower fear response to the CS− [2].

**Figure 1:**
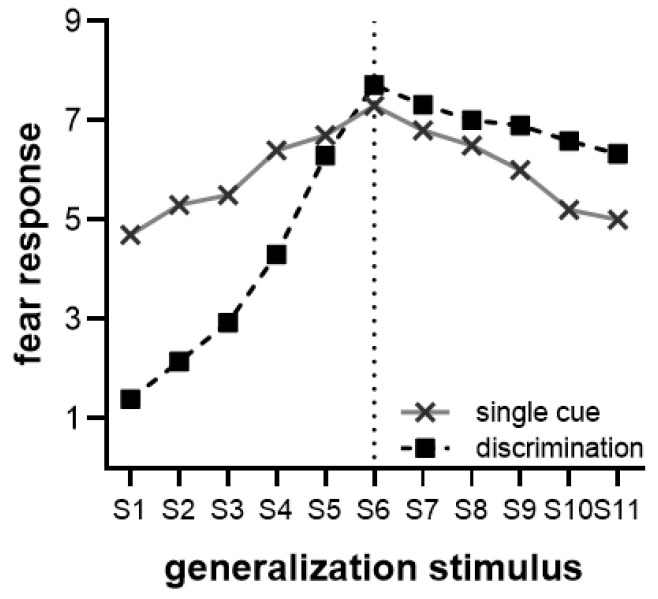
In the single cue condition, where stimulus (S) 6 was trained as the threat-conditioned stimulus (CS+), participants showed the highest fear response to S6, with elevated fear responses observed for the nearby stimuli (S4, S5, S7, and S8) during the generalization test. Conversely, stimuli further away from S6 (S1, S2, S10, and S11) elicited lower levels of fear expectation, resulting in a quadratic trend overall. In the discrimination condition, where S1 was trained as the safe conditioned stimulus (CS−) and S6 as the CS+, a linear trend was observed from S1 to S6, with progressively stronger fear responses as the stimuli approached S6. Adjacent stimuli to the CS− (S2, S3) elicited weaker fear responses.

In fear generalization, novel stimuli that are similar to the safe cue elicit weaker fear responses because the safe cue inhibits fear expression. Novel stimuli that are similar to the threat cue, by contrast, elicit a stronger fear response [2,8]. Different training conditions result in varying fear responses to untrained GS. Specifically, in the single cue condition, an overall quadratic trend was observed for a peak generalization gradient at CS+, with stimuli closer to CS+ eliciting stronger fear responses than stimuli farther away. In the discrimination condition, an overall strong linear trend was observed for the generalization gradient, with the lowest point at CS− and a peak at CS+ (see Figure 1 for an example). This suggests that adding a safe cue to the discrimination condition resulted in suppression of the fear response to novel stimuli around CS−, leading to a weaker generalization response compared with the single cue condition [6,7].

### 1.2. Rule-Based Fear Generalization

Fear can be generalized based on perceptual similarity and advanced semantic relationships, such as category knowledge [9,10], symbolic relationships [11], verbal instructions [12], classification, and inductive reasoning [13,14,15]. A key aspect is how the individual’s cognitive rules affect the generalization of fear. The use of different cognitive rules for stimuli varying in a single perceptual dimension produces different gradients of generalization. The linear rule and the similarity rule represent two distinct cognitive strategies or decision-making processes. The linear rule involves applying a linear relationship or progression to make judgments or predictions, resulting in a significant linear trend in the generalization gradient. This trend signifies a systematic pattern or direction of change that follows a linear relationship. In contrast, the similarity rule involves categorizing or judging stimuli based on their perceived similarity to a known reference stimulus [2]. When individuals use the similarity rule, the generalization gradient exhibits a significant quadratic trend [6,7], indicating a nonlinear relationship between the two variables.

Peak shift is an important phenomenon in the study of fear generalization. A peak represents the strongest fear response associated with a specific stimulus feature or location. Peak shifts occur when the maximum of a conditioned response moves in the direction of a specific attribute or feature that is similar—but not identical—to the initially learned stimulus. In discrimination learning, stimuli located near CS+ but away from CS− elicit maximal responses [6]. Lee and Livesey further explored rule-based generalization in humans in conditions with more complex rules. In the experiment, participants in the congruent group were allowed to use the rules learned in the previous stage for the discrimination of stimuli in the generalization test stage. In contrast, rules were changed for the incongruent group during the generalization test stage so that they could not use the rules previously acquired. The results showed a significant linear trend in the rule-based monotonic generalization gradient in the congruent group and a significant quadratic trend in the generalization gradient with peak shift in the incongruent group [16].

### 1.3. The Role of Context on Fear Generalization

Individual generalizations of fear are influenced by the nature of the stimulus itself, as with the single cue and discriminative conditioning paradigms and cognitive rules, and by contextual factors [17,18,19]. Variations in context alter the CS-US association, leading to the recovery of fear responses to a threat cue that had been extinct, as evidenced by increased amygdala activation [20], a greater skin conductance response (SCR) [21,22], an enhanced startle response [23], and higher fear ratings [22]. In AAB fear conditioning studies, fear responses that were acquired and extinct in context A were recovered in a new context B [24]. Furthermore, ABC contextual studies showed that after fear extinction in context B, the fear response recovered when switching to a new context C [22,24]. In the ABA context study, fear responses acquired in context A were found to be reactivated in context A after extinction in context B [25]. These findings indicate that fear learning involves both context and stimuli, with context performing a crucial role in maintaining fear [26] and being a decisive factor in the formation or extinction of the CS-US association [27].

Fear can generalize through contextual information. When participants were exposed to a new, harmless context that contained a mixture of safety and threat information, they exhibited stronger ratings of arousal, anxiety, and stress compared with safe environments [28]. Participants showed a strong fear response to a new situation that was similar to the original training situation [21]. Klein et al. [29] conducted research on fear generalization by combining stimuli and contexts. During the acquisition stage, they set up two contexts, an office and a library, with the library serving as a dangerous context and the office as a safe context. A blue or yellow lamp served as the stimulus. When the blue lamp was presented together with the library, it was paired with a 95 dB noise stimulus of 1 s duration (US) with a 100% reinforcement schedule. The yellow lamp and the office (safe context) were never paired with the US. In the generalization test stage, lamps of three colors were presented on a perceptual continuum from threat to safety as new stimuli combined with both types of contexts. Outcomes showed a more significant disparity in threat ratings for the new stimuli in the threat context compared with the safe context, confirming that the generalization response to stimuli is influenced by context.

### 1.4. The Current Study

Research often describes the effects of stimuli and contexts on fear generalization in a disparate, non-integrated way. The current study combined stimuli and contexts to investigate the complex influences on fear generalization, taking into account the dynamic diversity of real-life situations. We concentrated on the gradient and variation of fear generalization under several conditions, including the effects of learning cues, cognitive rules, and context on fear generalization. In our study, circle size was manipulated as the single dimension of change in stimulus perception, and increasing the intensity of the stimuli would affect the linear trend of conditioned responses [30,31,32]. Therefore, in the discrimination training condition, we assigned half of the participants a minimal circle as the safe cue and the other half a larger circle as the safe cue, making it possible to observe the gradient of fear generalization based on stimulus intensity and interpret it more rationally by combining cue conditions and rules. The current study contributes to a greater understanding of generalizations about fear and human emotions and behaviors. Furthermore, it has implications for the treatment and management of fear-related mood disorders.

## 2. Materials and Methods

### 2.1. Participants

A total of 104 participants were enrolled in the study and randomly assigned to one of four experimental groups. Participants were fully informed about the study and gave their consent to participate. All participants were healthy adults, right-handed, had not taken part in fear emotion studies in the past six months, and had no history of mental illness. The analysis took place with data from 99 participants (80 females) because five participants were excluded for failing to learn the CS-US association. The average age was 20.31 years (standard deviation: 1.944).

### 2.2. Apparatus and Stimuli

**Figure 2:**
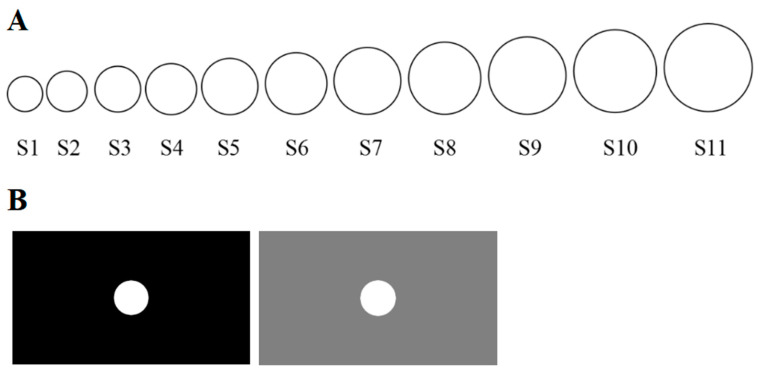
(**A**) Circles of different diameters were used as the conditioned (CS) or generalization stimuli (GS). (**B**) Two contexts—black and gray—were investigated.

The experiment was run on a computer using EPRIME 2.0. The CS and GS were 11 circle stimuli of different sizes (Figure 2A). The smallest circle had a diameter of 5 cm, while the largest one’s diameter was 12.5 cm. The other circles progressively increased in diameter by 15% from smallest to largest. We chose two different backgrounds, black and gray, to provide two different contexts (Figure 2B).

The Digitimer DS2A constant current stimulator (Hertfordshire, UK) was attached to participants’ right wrists with disposable electrodes during the experiment, and the stimulator produced 500 ms of shock as the unconditioned stimulus (US). The intensity of the shock was individualized for each participant. Before the formal experiment, participants rated their shock tolerance on a scale of 1 to 9, and each participant’s rating of 8 (i.e., ‘very uncomfortable but still tolerable’) was eventually chosen as the US for the formal experiment [33].

### 2.3. Procedure

We randomly assigned the 99 participants into four groups, and a one-way ANOVA revealed no significant differences in age (*F*(3, 96) = 0.394, *p* = 0.758) or gender (*F*(3, 96) = 1.150, *p* = 0.333) between the four groups. Participants were informed and consented to the potential shocks before the experiment. The formal experiment included two stages: acquisition and generalization testing (see Table 1). It started with a red fixation point ‘+’ in the center of the screen for 500 ms, followed by an 8000 ms stimulus. The participants were asked to rate their level of fear expectation for the stimulus using a numeric keypad ranging from 1 (completely impossible) to 9 (completely possible) in the text that was simultaneously displayed beneath the stimulus. The text vanished after a response was entered. Then, a shock or a blank screen was presented for 500 ms. The time between trials varied from 13–17 s, with a mean time of 15 s [2].

In the acquisition stage, a circle stimulus was presented in the center of a black context. The single cue condition was only presented with one type of stimulus (the circle indicated in Figure 2A as S6) with a 75% reinforcement schedule shock (US) as the CS+. The discrimination condition involved two CSs: S6 with a 75% reinforcement schedule shock (US) as the CS+, then S1 without shock (US) as the CS− (for half the participants), and S11 without shock (US) as the CS− (for the other half). In the generalization test stage, we presented 11 stimuli in random order. The consistent context was ensured by continuing use of the black background, and inconsistent context meant the use of a gray background. The experiment was approved by the ethics committee of South China Normal University (SCNU-PSY-2021-206).

### 2.4. Questionnaire

After the experiment, participants were asked to complete a questionnaire regarding the rules they used to identify the fear expectancy of stimuli in the generalization test phase. Participants were required to choose one of three options: linearity, similarity, or no rules.

### 2.5. Data Analysis

During the acquisition stage, in the single cue group, we conducted a two-factor mixed ANOVA with phases (within subjects) and contexts (between subjects) to examine the fear expectancy ratings of the CS+ over time and compare the acquisition differences between consistent and inconsistent contexts. In the discrimination group, a three-factor mixed ANOVA was conducted with stimulus type (within subjects) × trial (within subjects) × context to investigate the discrimination learning differences between the CS+ and CS− across trials and examine the impact of context on fear responses.

For the generalization test stage, we determined the peak of the generalization gradient by finding the stimulus that elicited the highest mean US expectation under specific conditions. To confirm the peak, we conducted *t*-tests comparing this stimulus with S1 and with S11. Additionally, we performed a *t*-test between the stimulus that elicited the highest US expectation and the CS+ to assess if there was a significant peak shift. Furthermore, we performed a three-factor mixed ANOVA with stimulus type × cue (between subjects) × context to examine the effect of the cue conditions and contexts on fear expectancy. We also performed a four-factor mixed ANOVA with the stimulus type × cue × rule × context to understand the main effects of each factor and the interaction effects between factors during fear generalization. Finally, a three-factor mixed ANOVA was performed with stimulus type × safety type (between-subjects) × rule to investigate the effect of stimulus intensity on fear generalization.

## 3. Results

### 3.1. Acquisition

#### 3.1.1. Single Cue Condition

**Figure 3:**
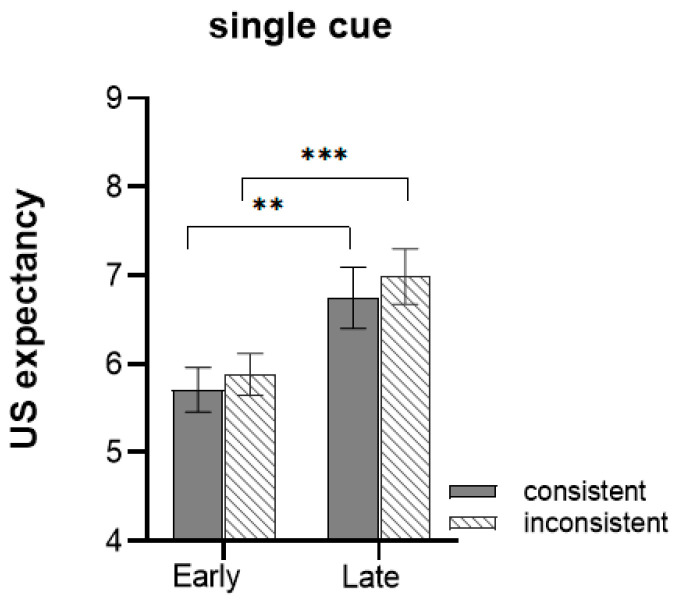
The mean unconditioned stimulus (US) expectancy ratings were higher for the late acquisition phase than the early phase. There was no significant difference between the two types of context groups. Error bars represent one standard error of the mean. *** *p* < 0.001, ** *p* < 0.01.

Results for the acquisition stage are summarized in Figure 3. The expected US ratings for CS+ on trials 1–3 were classified as the early phase, while trials 4–6 were classified as the late phase. The mixed ANOVA conducted with phase (early, late) × context (consistent, inconsistent) showed a significant main effect of time, *F*(1, 48) = 27.960, *p* < 0.001, $\eta_{p}^{2}$ = 0.368. The US expectancy ratings were higher in the late phase than in the early phase. As the training progressed, participants in the late acquisition phase successfully developed fear expectations for the CS+. We found no main effect of context, *F*(1, 48) = 0.003, *p* = 0.956, $\eta_{p}^{2}$ < 0.001, and no interaction with the phase.

#### 3.1.2. Discriminative Condition

**Figure 4:**
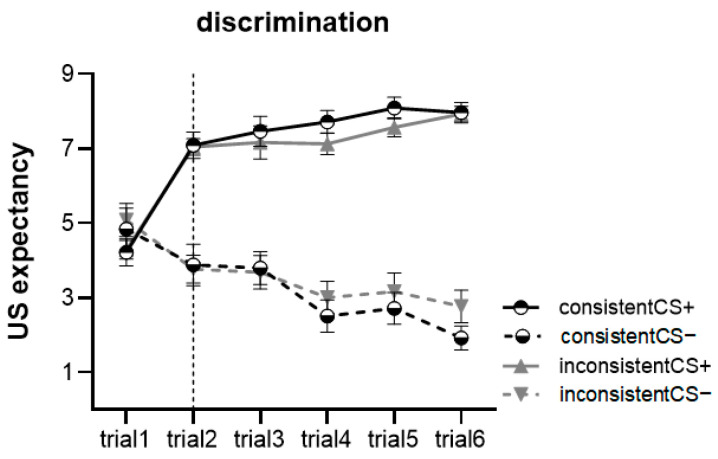
Both types of context groups had higher mean US expectancy ratings for CS+ than CS−. Since trial 2, participants have learned the difference between the CS-US association of CS+ and CS−. Error bars represent one standard error of the mean.

Figure 4 summarizes the results for the discriminative condition. The three-way mixed ANOVA was conducted with stimulus type (CS+, CS−) × trial (trial 1-trial 6) × context (consistent, inconsistent). Results showed a significant main effect of stimulus type, *F*(1, 47) = 127.852, *p* < 0.001, $\eta_{p}^{2}$ = 0.731, indicating that participants reported significantly higher US expectancy ratings for CS+ than for CS−. There was also a significant main effect of the trial, *F*(5, 43) = 8.169, *p* < 0.001, $\eta_{p}^{2}$ = 0.487, participants discerned differences in the type of CSs in trial 2. Moreover, there was a significant interaction effect between stimulus type and trial, *F*(1, 43) = 37.259, *p* < 0.001, $\eta_{p}^{2}$ = 0.812. We found no main effect of context, *F*(1, 47) = 0.016, *p* = 0.898, $\eta_{p}^{2}$ < 0.001, and no interaction with any other factors.

### 3.2. Generalization

We observed an overall significant linear trend (*F*(1, 95) = 131.945, *p* < 0.001, $\eta_{p}^{2}$ = 0.581), and a significant quadratic trend (*F*(1, 95) = 47.657, *p* < 0.001, $\eta_{p}^{2}$ = 0.334) in the mean of participants’ US expectancy ratings across the four groups. Additionally, we found an interaction effect between linear trend and cue, *F*(1, 95) = 8.176, *p* = 0.005, $\eta_{p}^{2}$ = 0.079, while no significant interaction effects were found between quadratic trend and cue, and there was no interaction effect between gradient trend and context.

#### 3.2.1. Generalization Based on Training Cues

**Figure 5:**
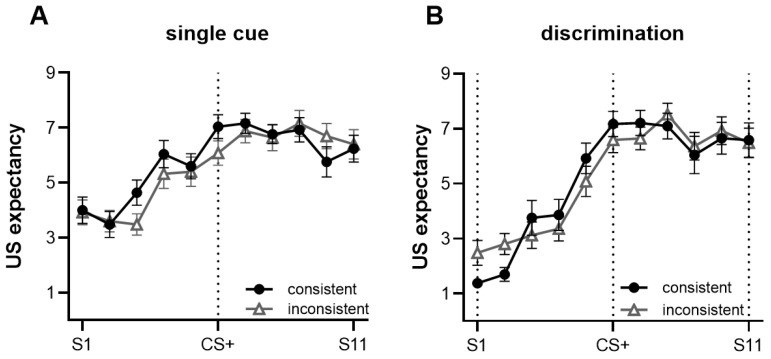
(**A**) The mean US expectancy ratings and generalization gradient in the single cue condition. (**B**) The mean US expectancy ratings and generalization gradient in the discrimination condition. The mean US expectancy ratings of all stimuli in the single cue condition were higher than the discrimination condition; specifically, S1, S2, and S4 had a significant difference between the two cue conditions. Error bars represent one standard error of the mean.

In the single cue condition, comprising both contextually consistent and inconsistent groups trained with a single cue, we examined the peak shift and found that participants had the highest mean US expectancy ratings for S9, with a significant difference compared with S1 (*t*(49) = 6.918, *p* < 0.001, Cohen’s *d* = 0.988) and S11 (*t*(49) = 2.107, *p* = 0.040, Cohen’s *d* = 0.301). We observed a generalization gradient with the peak at S9. There was no significant difference between S9 and CS+, *t*(49) = 1.284, *p* = 0.205, Cohen’s *d* = 0.183, and the evidence for peak shift was weak. Furthermore, the generalization gradient showed a significant linear trend, *F*(1, 48) = 52.173, *p* < 0.001, $\eta_{p}^{2}$ = 0.521, and a significant quadratic trend, *F*(1, 48) = 14.758, *p* < 0.001, $\eta_{p}^{2}$ = 0.235 (see Figure 5A).

In the discrimination condition, comprising both contextually consistent and inconsistent groups trained to discriminate, we examined the peak shift and found that participants had the highest mean US expectancy ratings for S8, without significant differences compared with S11, *t*(48) = 1.754, *p* = 0.087, Cohen’s *d* = 0.253. There was no significant difference between S8 and CS+, *t*(48) = 0.921, *p* = 0.362, Cohen’s *d* = 0.133, indicating no peak shift. Moreover, the generalization gradient showed a significant linear trend, *F*(1, 47) = 79.253, *p* < 0.001, $\eta_{p}^{2}$ = 0.628, and a significant quadratic trend, *F*(1, 47) = 36.018, *p* < 0.001, $\eta_{p}^{2}$ = 0.434 (see Figure 5B).

Mixed ANOVA results (stimulus type (S1–S11) × cue (single, discrimination) × context (consistent, inconsistent)) showed a significant main effect of stimulus type, *F*(10, 86) = 26.545, *p* < 0.001, $\eta_{p}^{2}$ = 0.755, indicating that participants had different US expectancy ratings for the stimuli. A significant main effect of the cue, *F*(1, 95) = 5.310, *p* = 0.023, $\eta_{p}^{2}$ = 0.053, suggested that there were higher US expectancy ratings in the single cue condition than the discrimination condition during the generalization testing stage. In addition, there was a significant interaction effect between stimulus type and cue, *F*(10, 86) = 5.902, *p* < 0.001, $\eta_{p}^{2}$ = 0.407. Simple effect analyses revealed that the single cue condition had a higher US expectancy rating than the discrimination condition for S1 (*F*(1, 95) = 24.293, *p* < 0.001, $\eta_{p}^{2}$ = 0.204), S2 (*F*(1, 95) = 11.272, *p* = 0.001, $\eta_{p}^{2}$ = 0.106), and S4 (*F*(1, 95) = 105.951, *p* < 0.001, $\eta_{p}^{2}$ = 0.146). There was no main effect of context, *F*(1, 95) = 2.383, *p* = 0.653, $\eta_{p}^{2}$ = 0.002, and no significant interaction effect between context and cue, *F*(1, 95) = 2.413, *p* = 0.205, $\eta_{p}^{2}$ = 0.002.

#### 3.2.2. Generalization Based on Rule

**Figure 6:**
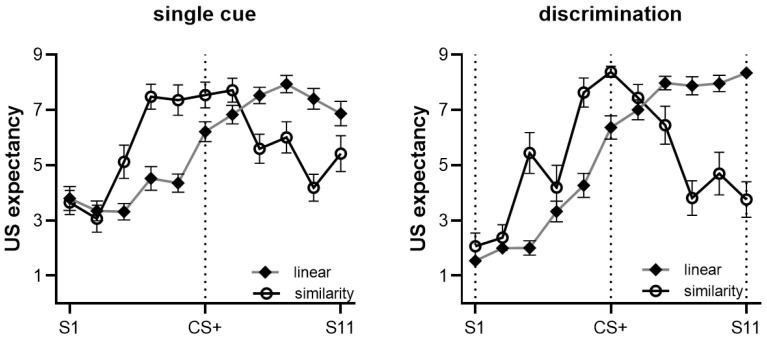
The generalization gradient of linear and similarity rules in the two cue training conditions. In the single cue condition, the linear rule gradient showed the peak shift at S9, while the similarity rule gradient had a ‘flat’ gradient around S4–S8. In the discrimination condition, the linear rule gradient showed the peak shift at S11, and the similarity rule gradient showed a ‘convex’ gradient around S4–S8. Error bars represent one standard error of the mean.

Participants were divided into ‘linear rule’ (*n* = 59), ‘similarity rule’ (*n* = 33), or ‘no rule’ (*n* = 7) groups based on their post-test questionnaires. We excluded participants with no rule from the analysis. Thus, a total of 92 participants were included in the following analysis, and no significant age (*F*(1, 89) = 0.188, *p* = 0.666) or gender (*F*(1, 89) = 2.022, *p* = 0.159) differences were found between the groups. The findings for the linear and similarity rule groups are presented in Figure 6 and discussed in further detail below.

**Linear rule**. We examined the peak of the generalization gradient for participants who adopted the linear rule. The results showed that S9 had the highest US expectancy ratings (S9 vs. S11, *t*(58) = 1.074, *p* = 0.287, Cohen’s *d* = 0.141). No gradient peak was found on S9, but there was a significant difference between S9 and CS+, *t*(58) = 5.280, *p* < 0.001, Cohen’s *d* = 0.693, indicating that participants who used the linear rule tended to generalize US expectations for the larger stimuli. There was a significant linear trend, *F*(1, 55) = 413.540, *p* < 0.001, $\eta_{p}^{2}$ = 0.883, and a significant quadratic trend, *F*(1, 55) = 13.199, *p* = 0.001, $\eta_{p}^{2}$ = 0.194. Furthermore, there was a significant interaction effect between the linear trend and the cue, *F*(1, 55) = 23.590, *p* < 0.001, $\eta_{p}^{2}$ = 0.300, but no interaction effect between the quadratic trend and cue. We examined peak shift in the single cue group and found that S9 had the maximum rating of the US expectancy: S9 vs. S1, *t*(28) = 8.147, *p* < 0.001, Cohen’s *d* = 1.540, and S9 vs. S11, *t*(28) = 2.880, *p* = 0.008, Cohen’s *d* = 0.544. The peak of the gradient was at S9: S9 vs. CS+, *t*(28) = 5.185, *p* < 0.001, Cohen’s *d* = 0.980, with the peak shifting from CS+ to S9. In the single cue condition, those who adopted the linear rule showed a significant linear trend, *F*(1, 28) = 95.096, *p* < 0.001, $\eta_{p}^{2}$ = 0.773, but no significant quadratic trend, *F*(1, 28) = 2.951, *p* = 0.097, $\eta_{p}^{2}$ = 0.095. In the discrimination group, S11 had the maximum rating of the US expectancy, S11 vs. S1, *t*(29) = 26.157, *p* < 0.001, Cohen’s *d* = 4.857. The largest circle, S11, was the peak of the gradient (S11 vs. CS+, *t*(29) = 4.595, *p* < 0.001, Cohen’s *d* = 0.853), with the peak shifting from CS+ to S11. There was a significant linear trend, *F*(1, 29) = 468.264, *p* < 0.001, $\eta_{p}^{2}$ = 0.942, and a significant quadratic trend, *F*(1,29) = 9.841, *p* = 0.004, $\eta_{p}^{2}$ = 0.253.

**Similarity rule.** We found CS+ had the highest US expectancy rating. We examined peak shift, CS+ vs. CS−, *t*(32) = 9.626, *p* < 0.001, Cohen’s *d* = 1.701, and CS+ vs. S11, *t*(32) = 5.636, *p* < 0.001, Cohen’s *d* = 0.996. The peak of the gradient occurred at CS+, and no peak shift was noted. The gradient had a significant linear trend, *F*(1, 29) = 10.720, *p* = 0.003, $\eta_{p}^{2}$ = 0.270, and a significant quadratic trend, *F*(1, 29) = 88.384, *p* < 0.001, $\eta_{p}^{2}$ = 0.753. We analyzed the five stimuli (S4–S8) surrounding CS+, finding a significant interaction of linear trend and cue, *F*(1, 29) = 5.778, *p* = 0.023, $\eta_{p}^{2}$ = 0.166, and quadratic trend and cue, *F*(1, 29) = 7.717, *p* = 0.009, $\eta_{p}^{2}$ = 0.210. Further analysis showed that the single cue group had a significant linear trend, *F*(1, 16) = 5.734, *p* = 0.029, $\eta_{p}^{2}$ = 0.264, and a significant quadratic trend, *F*(1, 16) = 8.150, *p* = 0.011, $\eta_{p}^{2}$ = 0.337. The discrimination group did not show a significant linear trend, only a significant quadratic trend, *F*(1, 15) = 33.747, *p* < 0.001, $\eta_{p}^{2}$ = 0.692. The results suggest that participants who adopted the similarity rule produced a more convex generalization gradient to stimuli surrounding CS+ for the discrimination group compared with the single cue group.

**Figure 7:**
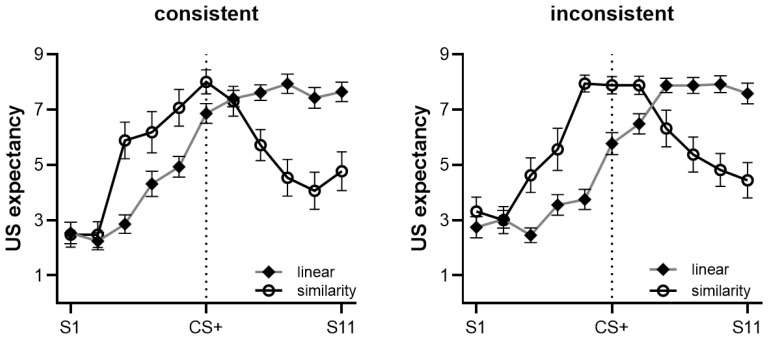
The generalization gradient of the linear rule in the consistent context condition showed a significant linear trend and a significant quadratic trend. In the inconsistent context condition, only a significant linear trend was noted. Error bars represent one standard error of the mean.

**Interaction of gradient trend and context.** We analyzed the gradient trends between participants using the linear rule in consistent context and inconsistent context conditions. It revealed a significant interaction between quadratic trend and context, *F*(1, 55) = 6.899, *p* = 0.011, $\eta_{p}^{2}$ = 0.111. Specifically, in the consistent context condition, participants using the linear rule exhibited both a significant linear trend (*F*(1, 26) = 406.100, *p* < 0.001, $\eta_{p}^{2}$ = 0.940) and a significant quadratic trend (*F*(1, 26) = 14.601, *p* = 0.001, $\eta_{p}^{2}$ = 0.360). In contrast, in the inconsistent context condition, participants using the linear rule demonstrated a significant linear trend, *F*(1, 29) = 153.106, *p* < 0.001, $\eta_{p}^{2}$ = 0.841, while the quadratic trend showed a non-significant result (*F*(1, 29) = 0.709, *p* = 0.407, $\eta_{p}^{2}$ = 0.024). By comparing participants using the similarity rule in the consistent context and inconsistent context conditions, we found no significant difference in their gradient trends (see Figure 7).

**Interaction of rule, cue, and context**. We performed a four-way mixed ANOVA on stimulus type (S1–S11) × rule (linear, similarity) × cue (single, discrimination) × context (consistent, inconsistent). The results showed the main effect of stimulus type was significant, *F*(10, 75) = 35.043, *p* < 0.001, $\eta_{p}^{2}$ = 0.824, and the main effect of cue was also significant, *F*(1, 84) = 54.893, *p* = 0.031, $\eta_{p}^{2}$ = 0.054. However, there was no significant main effect of rule, *F*(1, 84) = 1.069, *p* = 0.761, $\eta_{p}^{2}$ = 0.001, nor a significant main effect of context, *F*(1, 84) < 0.001, *p* = 0.987, $\eta_{p}^{2}$ < 0.001. We found a significant interaction of stimulus type × rule × cue, *F*(10, 75) = 3.301, *p* = 0.001, $\eta_{p}^{2}$ = 0.305.

#### 3.2.3. Generalization Based on Stimulus Intensity

**Figure 8:**
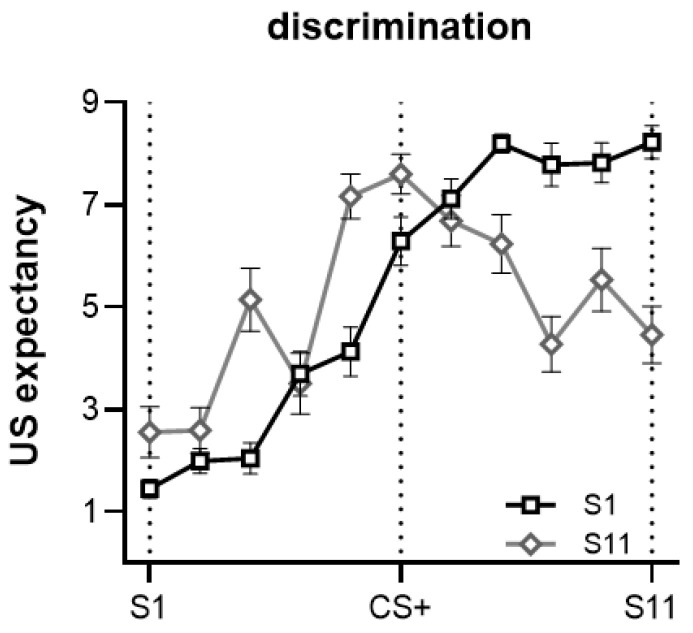
The peak shift and a main linear trend when using S1 as a safe cue; a main quadratic trend and no peak shift when using S11 as a safe cue. Error bars represent one standard error of the mean.

**S1 was the safe cue**. We found the maximum expected of the US for S11, and examined S11 vs. S1, *t*(26) = 17.898, *p* < 0.001, Cohen’s *d* = 3.510. S11 was the peak of the generalization gradient. S11 vs. CS+, *t*(26) = 3.477, *p* = 0.002, Cohen’s *d* = 0.681, suggested a significant peak shift from the CS+ to S11. A significant linear trend from S1 to S11 was noted, *F*(1, 26) = 266.921, *p* < 0.001, $\eta_{p}^{2}$ = 0.911, along with a significant quadratic trend, *F*(1, 26) = 8.146, *p* = 0.008, $\eta_{p}^{2}$ = 0.239 (Figure 8).

**S11 was the safe cue**. We found the maximum expected of the US for CS+, and examined CS+ vs. S1, *t*(18) = 12.004, *p* < 0.001, Cohen’s *d* = 2.829, CS+ vs. S11, *t*(18) = 4.991, *p* < 0.001, Cohen’s *d* = 1.176. The generalization gradient has a peak at CS+ without a significant peak shift. We found a significant linear trend, *F*(1, 18) = 12.768, *p* = 0.002, $\eta_{p}^{2}$ = 0.415, and a significant quadratic trend, *F*(1, 18) = 58.830, *p* < 0.001, $\eta_{p}^{2}$ = 0.766.

Finally, we analyzed the difference in US expectancy ratings and their interaction with the rule for two types of safe cues of different stimulus intensities in the discrimination condition. A three-way mixed ANOVA of stimulus type (S1–S11) × safety type (S1, S11) × rule (linear, similarity) revealed a significant main effect of stimulus type, *F*(10, 33) = 23.317, *p* < 0.001, $\eta_{p}^{2}$ = 0.876, and a significant interaction between stimulus type and safety type, *F*(10, 33) = 2.455, *p* = 0.026, $\eta_{p}^{2}$ = 0.427. No three-way interaction effect between stimulus type, safety type, and rule was found.

## 4. Discussion

Our study aimed to investigate the influence of cues and contexts on conditioned fear and the role of rule in shaping fear generalization across four distinct conditions. Specifically, the investigation centered on the distinctions in fear generalization performance among participants who underwent distinct cue training and encountered diverse contextual conditions. We examined changes in the gradient of fear generalization induced by variations in stimulus intensity. Throughout the generalization phase, a divergence in generalization patterns and response magnitudes to stimuli was observed between participants who underwent single cue and discrimination training. Notably, those who underwent single-cue training exhibited a generally greater intensity of fear expectation responses, while those in the discrimination training condition demonstrated a more pronounced linear trend. Participants’ fear expectation responses to stimuli presented with inconsistent context were of similar magnitude to those presented in a consistent context, indicating that the inconsistent context did not diminish the fear expectation responses to the stimuli. In addition, peak shifts were observed among participants who used the linear rule, particularly in those who underwent discrimination training. In the discrimination training condition, we observed a strengthened linear trend when changes in stimulus intensity and discriminative cues were in the same direction, whereas a symmetric peak gradient was evident when the changes were in opposite directions.

### 4.1. The Role of Cue Training on Fear Generalization

Our experimental results were similar to those of previous studies with regard to the different types of cue training that we manipulated [6,7]. The generalization gradient of fear showed significant linear and quadratic trends overall. However, there was an inconsistent linear trend in the generalization gradient between the two types of cue training conditions, and the cue training that participants received influenced their fear expectancy of the stimuli [34]. Participants in the discrimination training condition showed weaker fear generalization than those in the single cue training condition.

Perceptual discrimination may serve as a potential explanatory mechanism for the observed weaker fear generalization in the discrimination training paradigm. Through discrimination training, participants were able to differentiate between CS+ and CS− stimuli and become more sensitive to the differences between them. This increased sensitivity to differences facilitates the application of learned information to new stimuli, resulting in more precise reactions [35]. Moreover, this discrimination ability enables a better understanding of the differences between safety and threat signals and which stimuli are most threatening [36]. The ability to discriminate may have resulted in weaker fear generalization because participants were better able to identify stimuli that were genuinely threatening [37]. In contrast, during single cue training, participants solely focused on learning a single stimulus, making them less likely to perceive subtle differences between that stimulus and other stimuli. Consequently, they may respond less accurately to new stimuli. Additionally, participants may remain highly vigilant to all stimuli around them, resulting in stronger fear generalization because single cue learning does not distinguish between safety and threat signals.

### 4.2. The Role of Rule on Fear Generalization

In the current study, we observed a peak shift in participants who adopted the linear rule despite not showing the strongest fear response to the CS+. Instead, their fear expectations were highest for stimuli adjacent to CS+ [6]. Furthermore, we found that participants in the discrimination condition who employed the linear rule exhibited the greatest fear expectations for the biggest circle, indicating the most significant peak shift magnitude. The peak shift phenomenon arises because of the interplay between the excitatory nature of the CS+ and the inhibitory nature of the CS−. GSs located closer to the CS+ but further away from the CS−tend to be more strongly impacted by the excitatory effects of the CS+ while experiencing less inhibition from the CS−. As a result of this specific location, these GSs have a higher likelihood of eliciting fear responses [36]. In discriminative conditions, participants may employ perceptual classification of the stimuli whereby the presentation of a small circle CS− results in higher ratings being assigned to the bigger circle, which is classified as a CS+ [38]. Their capacity to reason using linear rules was strengthened by their discriminative learning [7], which may also explain the absence of peak shifts in the gradient for participants who employed the similarity rule.

The strength of fear responses in participants who employed the similarity rule depended on the similarity between the stimuli and the original cue that triggered fear [2]. As a result, participants who adopted the similarity rule presented a symmetrical generalization gradient that peaked at CS+. Remarkably, participants who received single cue training and adopted the similarity rule appeared unable to discriminate the true threat stimulus during the generalization test phase, presenting a flat generalization gradient. That finding, again, verified the effect of cue training on fear generalization. When only one threat cue was presented, the lack of learning of the safe cue failed to allow participants to form inferences about the safe and threat patterns of the stimulus. Therefore, they were limited to relying on the perception of the circle size for discrimination or memory. The potential threat situation created pressure that led to errors in participants’ memory of the original threat cue [39], making the localization inaccurate. At this point, participants exposed to the new stimulus were very uncertain and fear generalization was induced [40].

### 4.3. The Effect of Context on Fear Generalization

The current study found no changes in the overall generalization gradient across contexts. Further, we found no context-induced differences in fear generalization in the single-cue or discrimination training conditions, with only weak evidence of a quadratic trend effect of context on the fear generalization gradient observed in participants who used linear rules. Generally, both consistent (black) and inconsistent (gray) contexts showed equivalent levels of fear generalization, with the consistent context not reflecting stronger fear expectations compared with the inconsistent context—different from previous work [21,29,41].

Stimulus generalization is a more dominant process than context generalization, explaining why changing the background color did not significantly reduce fear generalization. Participants were primarily attending to and generalizing based on the similarity of the stimuli rather than the context in which they were presented. Therefore, even if the background color changes, if the stimuli remain similar, fear generalization is likely to occur. This explanation is consistent with the idea that fear generalization is driven by the perceptual similarity between stimuli [2]. Alternatively, participants can generalize their learned fear responses to the context via the stimuli, resulting in a generalized fear expectation towards inconsistent context. Alternatively, prospective threats (stimuli) and neutral information (context) displayed concurrently on the screen were interpreted as threatening by the participants to prevent missing threat information [4,28]. This may explain why there were similar fear responses even though the context changed.

The role of task relevance should also be considered when interpreting the effects of emotional stimuli. Recent evidence shows that emotional facial [42,43,44] or body expressions [45] evoke a consistent and reproducible behavioral response, but their impact is contingent on their relevance to participants’ goals. Mancini et al. [42] conducted a study using two versions of a Go/No-go task to investigate the impact of emotional facial expressions on motor readiness and reaching arm movement accuracy. They found that these expressions only influenced performance when they were relevant to the task. Threatening expressions increased reaction times and omission errors compared with happy faces, but only when the emotional content was necessary for the correct response. In task-irrelevant conditions, where participants responded based on facial gender, differences between happy and threatening expressions disappeared. Hence, considering the role of context, it is possible that integrating context with task relevance could provide a richer understanding of fear generalization effects.

### 4.4. The Role of Stimulus Intensity on Fear Generalization

The perceptual stimuli used in the study had intensity features, with significant linear trends observed in the fear generalization gradient for both the single cue and discrimination conditions. The generalization gradient for the single cue condition was not as symmetrical as previously reported [6,7]. The dimension of stimulus variation, involving stimulus intensity, generated a monotonic generalization gradient [31] whereby fear responses were strengthened with increasing stimulus intensity. Participants discriminate threat cues by inferring shape size [32,46], considering the larger the size, the more likely it is to be dangerous.

In this study, stimulus intensity was linked to increased circle size. We observed peak shifts in participants using the linear rule, especially in the discrimination condition in response to the most intense stimulus. Using the minimum circle (S1) as the safe cue and the mid-sized circle (S6) as the threat cue, the CS-US association changed in the same direction as the stimulus intensity, jointly leading to a strong linear trend [30,32]. Participants exhibited the most fearful expectation for the largest circle (S11) rather than the original threat cue (S6). However, the reverse operation with the largest circle (S11) as a safe cue and the medium circle (S6) as a threat cue revealed no peak shift and no overall negative linear trend. The fear response at this point showed a symmetric gradient of generalization with CS+ as the peak, exhibiting an increasing fear response from S1 to CS+ and a diminishing fear response from CS+ to S11 rather than a diminishing linear trend from S1 to S11. The results demonstrated that fear expectancies towards the stimuli exhibited an enhancing effect as a function of stimulus intensity but did not show a consistent incremental decrease as intensity decreased. One potential explanation is that the directional changes in the perceptual dimension of the safe and threat cues were opposite to the changes in stimulus intensity. The increasing gradient of generalization from S1 to CS+ may be because of the increase in stimulus intensity, with participants prioritizing stimulus intensity (circle size increasing from small to large). The decreasing gradient from CS+ to S11 may be due to participants learning the decreasing CS-US association between the CS− and the CS+ during the acquisition stage [34], prioritizing the single-dimensional change of safe and threat cues at this point (circle size decreasing from large to small).

This study presented a more intricate fear generalization effect compared with previous studies, thus contributing to a deeper comprehension of human learning and generalization mechanisms. We identified differences in fear expectancy responses based on single cue and discrimination training conditions, providing insight into the formation and maintenance mechanisms of fear and anxiety disorders under different learning conditions. Specifically, we showed that using the similarity rule in the single cue condition resulted in equivalent fear expectations for new stimuli around the threat cue. In contrast, the linear rule employed in the discrimination condition reinforced fear responses away from the safe cue, revealing that training in providing safe cues prompted fearful expectations of neutral stimuli, and suggesting that the availability of a safe cue may weaken or reverse the effects of exposure treatment. Therefore, it is important to be cautious in selecting and using cues during exposure therapy to avoid the negative effects of cue training on treatment efficacy. In addition, differences in fear generalization responses across multiple experimental conditions suggest the need for more individualized and differentiated interventions during treatment to improve the effectiveness of treatment for specific phobias.

The study was limited by the stimulus width, and the stimulus intensity meant we observed a maximum fear expectation for the most intense stimulus, showing a monotonic linear trend. Future studies could attempt to expand the range of stimulus widths, possibly resulting in generalized gradients with peaks [47] and significant quadratic trends. More generally, studies could explore fear generalization gradients in a wider range of stimuli after controlling the differences in discriminative perceptual ability between individuals [37] to obtain more detailed explanations of the role of stimulus intensity on generalization. Additionally, integrating contextual information within task-relevant conditions holds promise for uncovering a more comprehensive understanding of fear generalization [42,44]—an avenue of inquiry to explore the intricate interplay between emotional stimuli, task demands, and contextual factors.

## 5. Conclusions

This study demonstrated different patterns of fear generalization across cue training conditions and cognitive rules. More specifically, the safe cue in the discrimination condition inhibited the expression of fear toward surrounding stimuli compared with the single cue condition. A significant peak shift was observed in the linear rule but not in the similarity rule. Learned fear responses to stimuli were generalized across contexts. Changes in the perceptual dimension of safety and threat cues, combined with variations in stimulus intensity during discrimination acquisition, produced two distinct trends in the generalization gradient: a strong linear trend when changes were congruent and a quadratic trend when changes were incongruent rather than a negative linear trend.

## Figures and Tables

**Table 1:** The management of the four groups included two stages: acquisition and generalization testing. In the acquisition stage, the single cue group was only presented with S6, while the discrimination group was shown in S6 and S1 (or S11). During the generalization stage, the black background was employed for the consistent context, and gray was used for the inconsistent context.

Group			Acquisition				Generalization		
		*n*	Cue		Trial	Context	Stimuli	Trial	Context
Single cue	consistent	25	CS+	S6	6 (4US)	black	All	11	black
	inconsistent	25	CS+	S6	6 (4US)	black	All	11	gray
Discrimination	consistent	24	CS+	S6	6 (4US)	black	All	11	black
			CS−	S1/S11	6				
	inconsistent	25	CS+ CS−	S6	6 (4US)	black	All	11	gray
				S1/S11	6				

## Data Availability

The data of this study are available from the corresponding author upon reasonable request.
